# Stress Avulsion Fracture of the Patellar Tendon Following Open Reduction and Internal Fixation of a Transverse Patella Fracture With Cannulated Screws

**DOI:** 10.7759/cureus.66590

**Published:** 2024-08-10

**Authors:** Aysha Rajeev, Saurav Krishnan, George Koshy, Mintu Mariam Baby, Kiran Singisetti

**Affiliations:** 1 Trauma and Orthopaedics, Gateshead Health Foundation National Health Service (NHS) Trust, Gateshead, GBR; 2 General Medicine, Gateshead Health Foundation National Health Service (NHS) Trust, Gateshead, GBR

**Keywords:** open reduction internal fixation, fracture patella, patella fracture, internal fixation, patella tendon, stress riser, avulsion fracture

## Abstract

A stress-avulsion fracture of the inferior pole of the patella is rare. We report a case of a 65-year-old woman who underwent open reduction and internal fixation for a transverse fracture of the patella using cannulated screws inserted from the inferior pole of the patella. Subsequently, the patient developed an avulsion fracture of the inferior pole of the patella due to a stress riser from the prominent screw head. The avulsion fracture was treated with open repair and augmentation using a cerclage wire, and the stress riser was eliminated by burying the screw head into the bone. The outcomes were satisfactory. Preventing implant-related stress risers during internal fixation of fractures requires diligent surgical techniques.

## Introduction

Avulsion fracture of the patellar tendon from the inferior pole of the patella is relatively rare, with patellar tendon injuries comprising only 3% of all knee extensor system injuries [1]. Early diagnosis and operative treatment of knee extensor mechanism injuries are crucial to restoring function, integrity, and strength [2]. To date, stress riser-induced avulsion fractures of the inferior pole of the patella following internal fixation for patella fractures have not been reported in the literature.

In this case report, we present a unique instance of an avulsion fracture of the distal pole of the patella caused by a stress riser following internal fixation of a transverse patellar fracture with cannulated screws. This case underscores the importance of meticulous surgical technique and implant placement to prevent such complications.

## Case presentation

**Figure 1:**
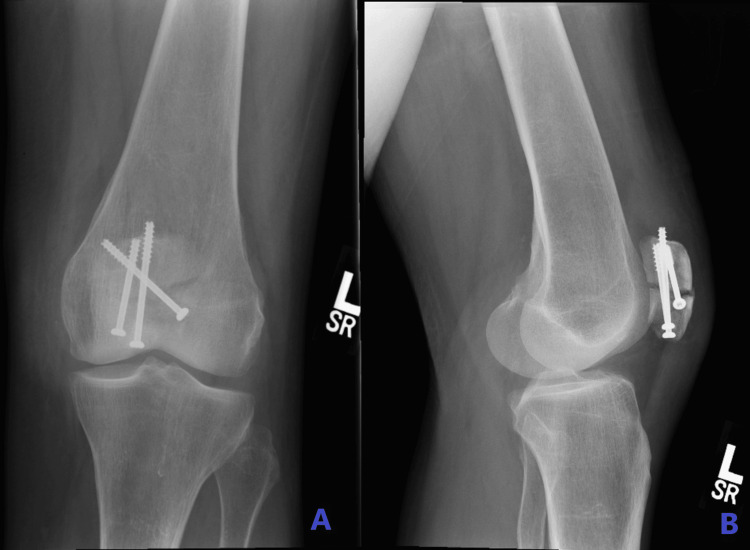
Plain antero-posterior (A) and lateral (B) X-rays after open reduction internal fixation of a transverse fracture of the patella

**Figure 2:**
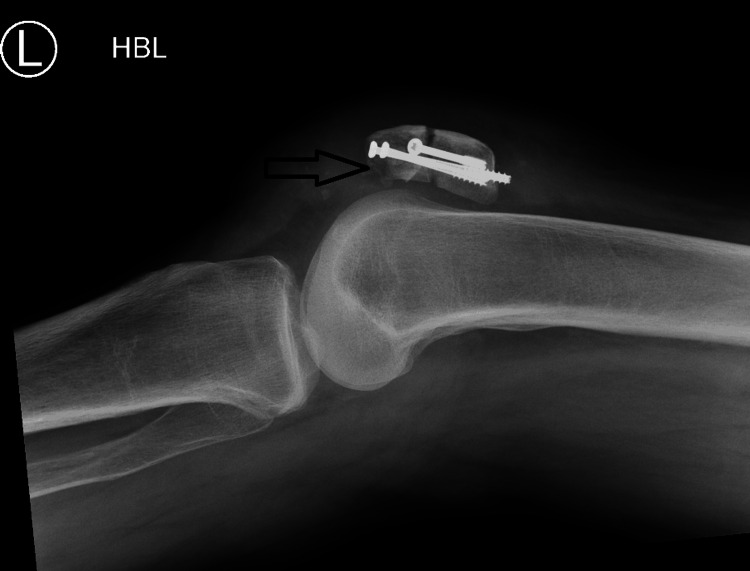
Lateral view of the knee showing stress avulsion fracture of the distal pole of the patella from the head of the previous screw fixation

**Figure 3:**
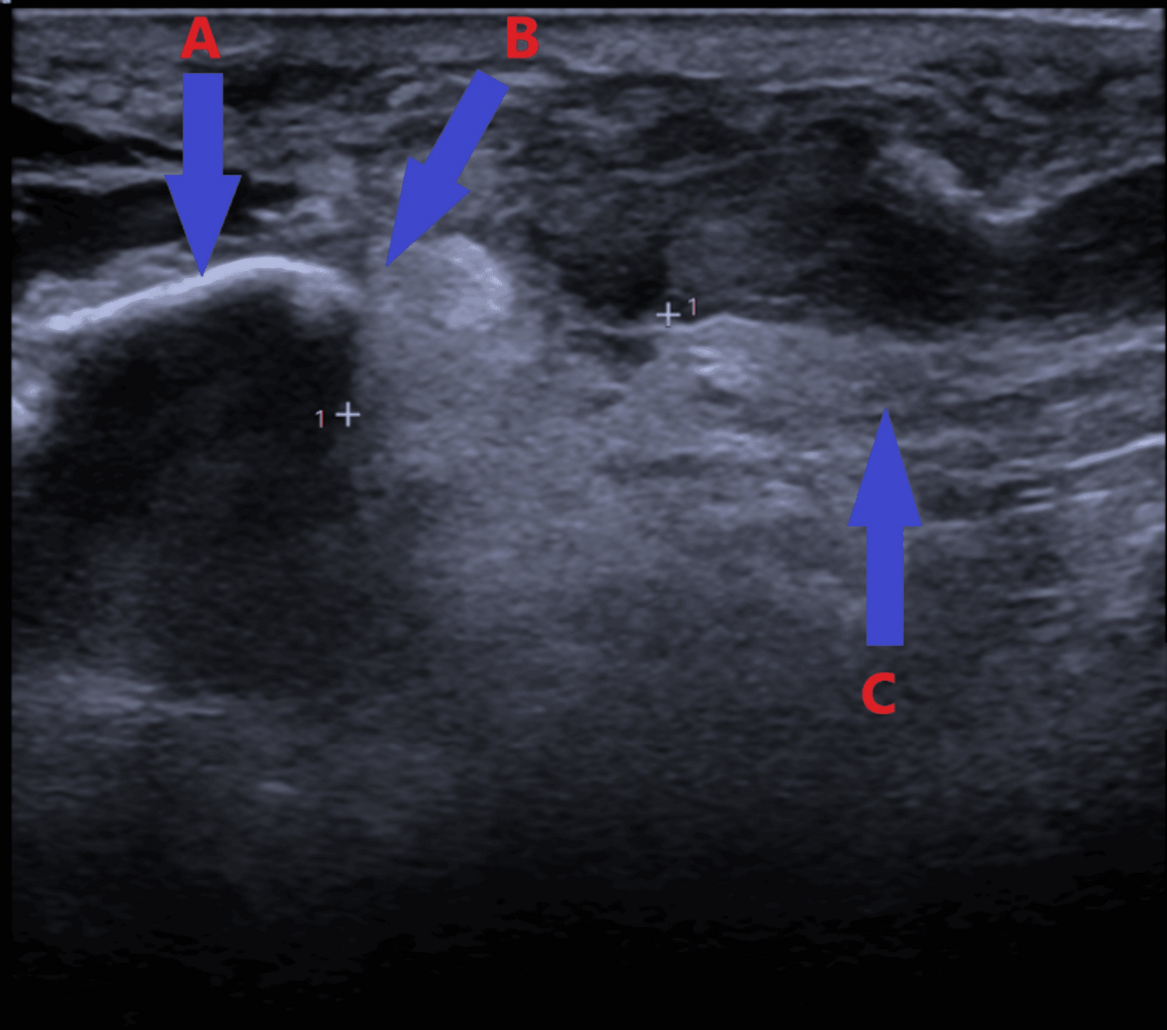
Ultrasound scan image showing avulsion of the patella tendon from the inferior pole of the patella: (A) patella, (B) avulsion fracture of the distal pole of the patella, and (C) patella tendon

A 65-year-old woman presented to our accident and emergency department with complaints of pain and swelling in her left knee and an inability to fully bear weight. She had fallen four weeks prior while on holiday, sustaining a transverse fracture of the patella, which was treated with open reduction and internal fixation using three cannulated screws (Figure 1A-1B). On examination in the emergency room, the patient exhibited gross knee swelling with effusion, tenderness at the distal pole of the patella, limited knee flexion to 20 degrees, and an inability to perform a straight leg raise. Her medical history included chronic obstructive pulmonary disease and osteoporosis, for which she was on oral bisphosphonates. Radiological examination revealed a stress avulsion fracture of the distal pole of the patella originating from the head of the previously inserted screw (Figure 2). Ultrasound imaging confirmed complete proximal patellar ligament disruption (Figure 3).

**Figure 4:**
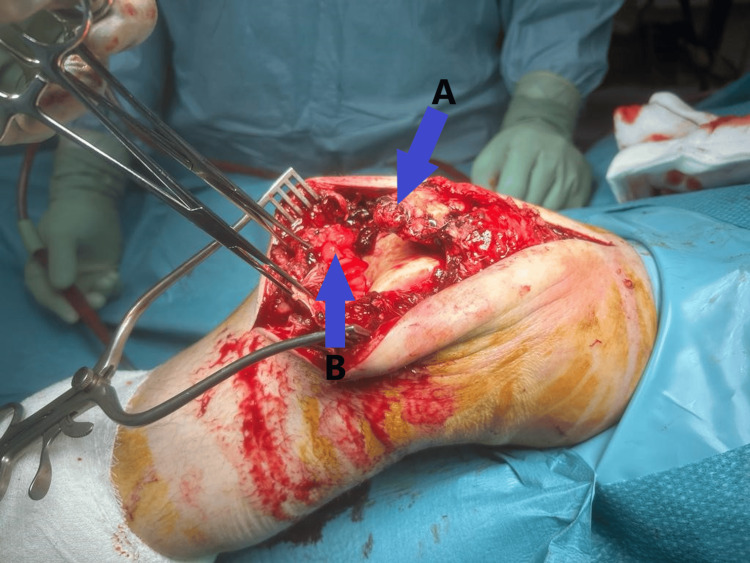
Intraoperative picture showing complete avulsion of the patella tendon from the inferior pole of the patella (A) and the ruptured end of the patella tendon (B)

**Figure 5:**
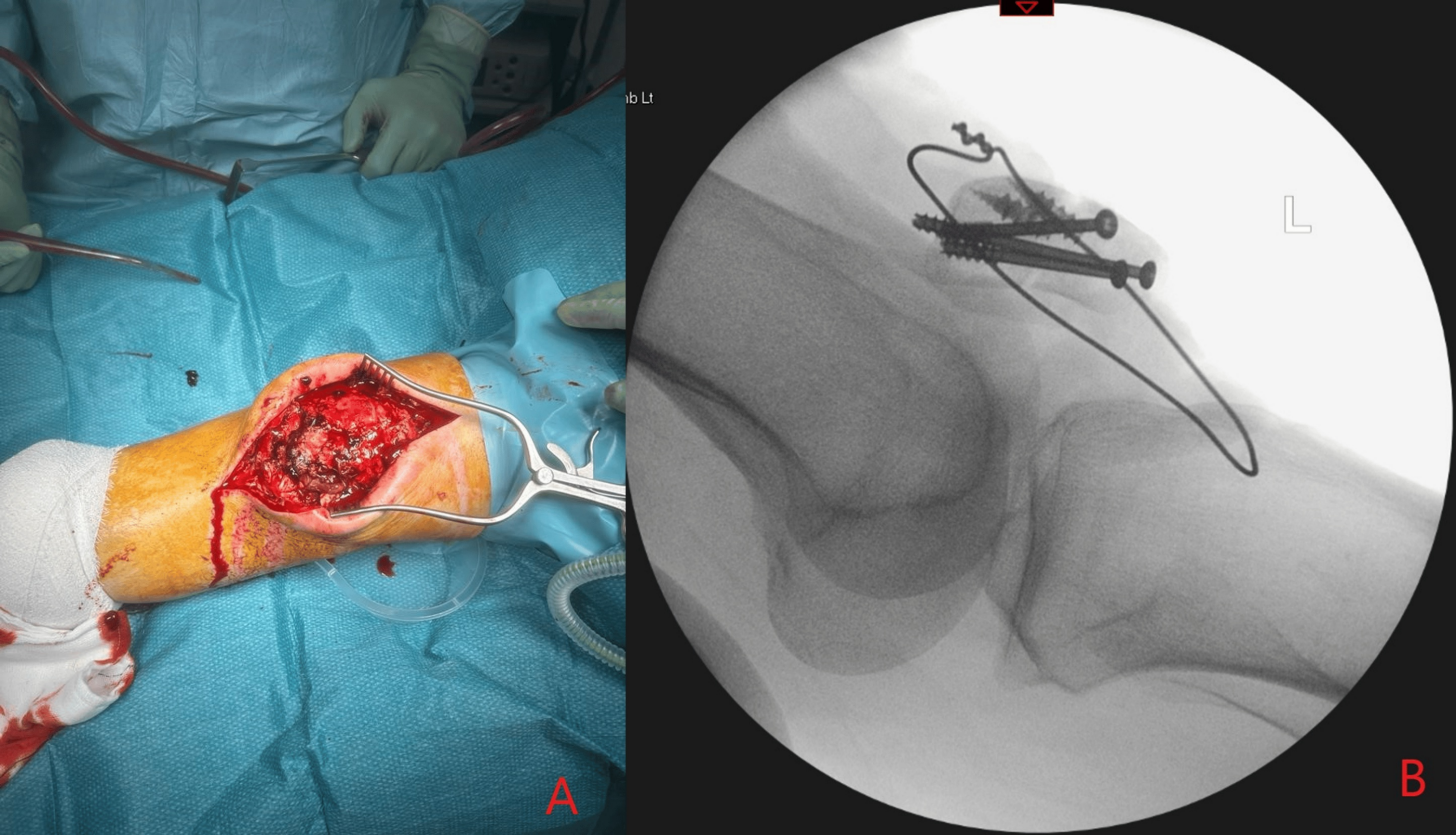
Intraoperative picture after the patella tendon repair (A) with image intensifier (B)

The patient consented to an open repair of the patellar tendon. Under general anesthesia, a midline incision was made on the anterior aspect of the knee. Surgical findings included a full-thickness rupture of the patellar tendon and retinaculum, with shredded tendon fibers (Figure 4). The cannulated screws were prominent but still secured the fracture site, so the decision was made to leave the fixation in situ after ensuring the screw heads were buried into the bone. The patellar tendon was prepared with Krackow sutures and reattached to the distal pole of the patella using two 5 mm titanium screw double-loaded anchors. A cerclage wire was placed around the patella and the tibial tuberosity to act as a de-tension device and protect the patellar tendon repair (Figure 5).

**Figure 6:**
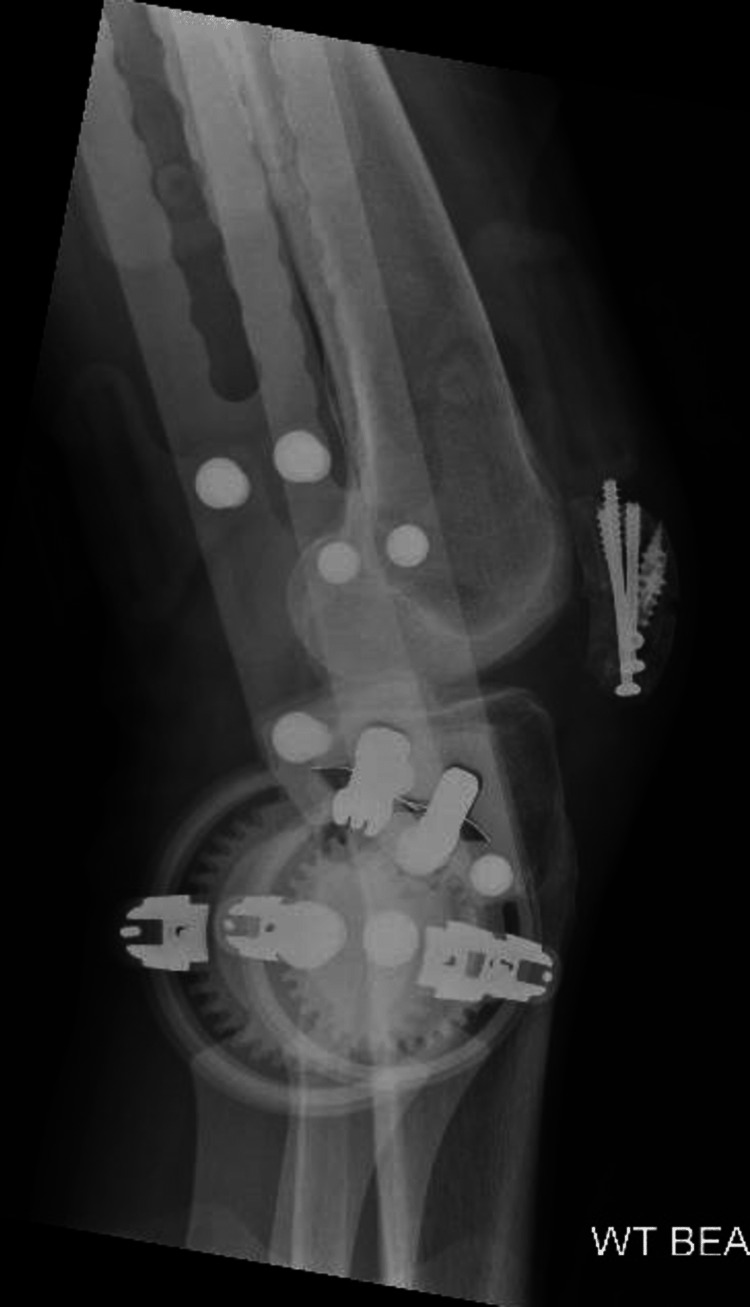
Lateral view of the knee showing healing of both the transverse and inferior pole fractures of the patella

The patient had an uneventful recovery and was followed up in the clinic at two weeks for wound inspection and suture removal. Her knee was immobilized in a cylinder cast for the first two weeks, followed by a hinged knee brace adjusted from 0 to 90 degrees of flexion for the next four weeks. After six weeks, the knee brace was removed and full range of motion exercises were initiated. The cerclage wire was removed five months post-surgery. At the final follow-up at six months, the patient had regained 90 degrees of knee flexion, was able to perform a straight leg raise, and could fully bear weight. X-ray examination showed that both the transverse and stress fractures of the distal pole of the patella had healed (Figure 6). She was subsequently discharged from the clinic.

## Discussion

The incidence of patella fractures in skeletally mature adults across all age groups is about 1% [3]. The occurrence of stress fractures of the patella is uncommon [4,5]. There are case reports of stress fractures of the patella following total knee replacement [6] and anterior cruciate reconstruction using patellar tendon bone grafts [7]. Other risk factors for patellar tendon ruptures include age over 40, repeated steroid injections [8], chronic patellar tendinopathy (especially in athletes) [9], diabetes, obesity, inflammatory arthropathy, and osteogenesis imperfecta. Systemic disorders such as hyperparathyroidism, chronic renal failure and dialysis, and Ehlers-Danlos syndrome also increase the risk of bilateral tendon injuries [10]. Long-term administration of fluoroquinolones has been associated with an increased risk of tendon ruptures [11].

The term "stress riser fracture," also known as Young's modulus fractures, was coined by Cameron et al. [12]. Stress riser fractures are caused by a concentration of stress in localized regions of the cortical bone and can lead to catastrophic failures. These fractures are often due to surgical technical errors and thus can be prevented. In our case, the stress riser was due to the prominent screw head used to internally fix the patella fracture. Over time, this resulted in an avulsion fracture of the distal pole of the patella.

Plain X-rays are often diagnostic for avulsion fractures of the patellar tendon, both from the inferior pole of the patella and the tibial tuberosity. Findings such as patella alta (high riding patella) and changes in the Insall-Salvati ratio (normal: 1-2) are classical signs on the lateral view of the knee [13]. Ultrasound examination is one of the most important imaging tools available for diagnosing patellar tendon ruptures. It is relatively inexpensive, quick, and easy to use. Ultrasound has the advantage of clearly evaluating and visualizing the fibrillar echotexture of normal tendons, and the superficial location of the patellar tendon makes it ideal for static and dynamic evaluation. Studies have shown that ultrasound examination is more accurate than MRI in evaluating patellar tendon injuries [14,15]. Our patient's ultrasound scan showed complete proximal patellar ligament disruption.

Partial tears of the patellar ligament can be treated non-operatively with an extension brace, gradually increasing flexion over a few weeks [16]. Open repair is the standard treatment of choice for complete ruptures and avulsion fractures. Transosseous repair is the most common technique used [17]. Capiola et al. described a suture anchor fixation to the inferior pole of the patella using a Krackow technique [18]. Augmentation of the repair can be done with metal cable wires, synthetic tapes, allografts, or autografts. The benefits of augmentation include decreased strain across the repair, which may result in earlier knee motion [16]. In our patient, the patellar tendon was reattached to the distal pole of the patella using two 5mm titanium screw double-loaded anchors after preparing the tendon with Krackow sutures, and was augmented with a cerclage wire around the patella and tibial tuberosity.

Post-operative rehabilitation varies from cast immobilization to the application of knee braces and early mobilization [19]. Most surgeons combine both principles: initial cast immobilization for the first few weeks followed by range-of-motion knee braces and protected weight-bearing. Our patient followed this combined rehabilitation protocol and achieved satisfactory outcomes regarding knee movements and weight-bearing.

## Conclusions

A stress fracture of the lower pole of the patella and avulsion of proximal patellar tendon due to prominent hardware is rare. In our case, the stress riser was caused by the prominent screw head from a previous patella fracture fixation. Early diagnosis and repair of the patellar tendon, along with correcting the factors leading to the stress riser, resulted in a good functional outcome. This case report highlights the importance of preventing stress risers caused by implants. Meticulous attention to surgical techniques during internal fixation of fractures can help avoid such complications.

## References

[1] Böhler B (1957). The treatment of fractures. J Bone Joint Surg Br.

[2] Vyas P, Cui Q (2020). Management options for Extensor Mechanism discontinuity in patients with total knee arthroplasty. Cureus.

[3] Posner AD, Zimmerman JP (2022). Surgical management of patella fractures: a review. Arch Orthop.

[4] Carneiro M, Nery CA, Mestriner LA (2006). Bilateral stress fracture of the patellae: a case report. Knee.

[5] Sillanpää PJ, Paakkala A, Paakkala T, Mäenpää H, Toivanen J (2010). Displaced longitudinal stress fracture of the patella: a case report. J Bone Joint Surg Am.

[6] Insall JN, Binazzi R, Soudry M, Mestriner LA (1985). Total knee arthroplasty. Clin Orthopaed.

[7] Stein DA, Hunt SA, Rosen JE, Sherman OH (2002). The incidence and outcome of patella fractures after anterior cruciate ligament reconstruction. Arthroscopy.

[8] Chen SK, Lu CC, Chou PH, Guo LY, Wu WL (2009). Patellar tendon ruptures in weight lifters after local steroid injections. Arch Orthop Trauma Surg.

[9] Monroy A, Urruela A, Egol KA, Tejwani NC (2013). Bilateral disruption of soft tissue extensor mechanism of knee: functional outcome and comparison to unilateral injuries. HSS J.

[10] Boublik M, Schlegel T, Koonce R, Genuario J, Lind C, Hamming D (2011). Patellar tendon ruptures in national football league players. Am J Sports Med.

[11] Daneman N, Lu H, Redelmeier DA (2015). Fluoroquinolones and collagen associated severe adverse events: a longitudinal cohort study. BMJ Open.

[12] Cameron HU, Pilliar RM, Hastings DE, Fornasier VL (1975). Iatrogenic subcapital fracture of the hip: a new complication of intertrochanteric fractures. Clin Orthop Relat Res.

[13] Verhulst FV, van Sambeeck JD, Olthuis GS, van der Ree J, Koëter S (2020). Patellar height measurements: Insall-Salvati ratio is most reliable method. Knee Surg Sports Traumatol Arthrosc.

[14] Warden SJ, Kiss ZS, Malara FA, Ooi AB, Cook JL, Crossley KM (2007). Comparative accuracy of magnetic resonance imaging and ultrasonography in confirming clinically diagnosed patellar tendinopathy. Am J Sports Med.

[15] Girish G, Finlay K, Landry D, O'Neill J, Popowich T, Jacobson J, Friedman L (2007). Musculoskeletal disorders of the lower limb-ultrasound and magnetic resonance imaging correlation. Can Assoc Radiol J.

[16] Golman M, Wright ML, Wong TT, Lynch TS, Ahmad CS, Thomopoulos S, Popkin CA (2020). Rethinking patellar tendinopathy and partial patellar tendon tears: a novel classification system. Am J Sports Med.

[17] Imbergamo C, Sequeira S, Bano J, Rate WR 4th, Gould H (2022). Failure rates of suture anchor fixation versus transosseous tunnel technique for patellar tendon repair: a systematic review and meta-analysis of biomechanical studies. Orthop J Sports Med.

[18] Capiola D, Re L (2007). Repair of patellar tendon rupture with suture anchors. Arthroscopy.

[19] West JL, Keene JS, Kaplan LD (2008). Early motion after quadriceps and patellar tendon repairs: outcomes with single-suture augmentation. Am J Sports Med.

